# Comparison of the Infectivity and Transmission of Contemporary Canine and Equine H3N8 Influenza Viruses in Dogs

**DOI:** 10.1155/2013/874521

**Published:** 2013-10-01

**Authors:** Heidi L. Pecoraro, Susi Bennett, Kristina Garretson, Ayshea M. Quintana, Katharine F. Lunn, Gabriele A. Landolt

**Affiliations:** ^1^Department of Microbiology, Immunology, and Pathology, College of Veterinary Medicine and Biological Sciences, Colorado State University, 300 West Drake Road, Campus Delivery 1678, Fort Collins, CO 80523, USA; ^2^Department of Clinical Sciences, College of Veterinary Medicine and Biological Sciences, Colorado State University, 300 West Drake Road, Campus Delivery 1678, Fort Collins, CO 80523, USA

## Abstract

Phylogenetic analyses indicate that canine influenza viruses (CIVs) (H3N8) evolved from contemporary equine influenza virus (EIV). Despite the genetic relatedness of EIV and CIV, recent evidence suggests that CIV is unable to infect, replicate, and spread among susceptible horses. To determine whether equine H3N8 viruses have equally lost the ability to infect, cause disease, and spread among dogs, we evaluated the infectivity and transmissibility of a recent Florida sublineage EIV isolate in dogs. Clinical signs, nasal virus shedding, and serological responses were monitored in dogs for 21 days after inoculation. Real-time reverse transcription-PCR and hemagglutination inhibition assays showed that both the viruses have maintained the ability to infect and replicate in dogs and result in seroconversion. Transmission of EIV from infected to sentinel dogs, however, was restricted. Furthermore, both CIV and EIV exhibited similar sialic acid-**α**2,3-gal receptor-binding preferences upon solid-phase binding assays. The results of the *in vivo* experiments reported here suggesting that dogs are susceptible to EIV and previous reports by members of our laboratory showing limited CIV infection in horses have been mirrored in CIV and EIV infections studies in primary canine and equine respiratory epithelial cells.

## 1. Introduction

Due to the partial host range restriction of influenza A viruses, transmission of an influenza virus from one species to another is relatively rare. However, such cross-species transmission events do occur and have generated severe disease outbreaks in new host species. The 1918 “Spanish flu” is a classic example of cross-species transmission with devastating results, as the influenza virus involved with the pandemic was likely transmitted directly from birds to humans [1]. Therefore, understanding the molecular mechanisms that allow these viruses to cross the species barrier and adapt to new hosts is crucial for identifying influenza viruses that could potentially threaten both human and animal health. While evidence has accumulated over the years indicating contributions by all eight gene segments [2–10], the examination of the impact of individual viral proteins to host range restriction is complicated by several factors. For example, mutations often occur in multiple gene segments during the process of virus adaptation to a new species [5, 11–13], and, while some of these mutations may indeed reflect adaptation of the virus to the new host, others may be introduced in response to host immune pressure, or they might simply represent spurious mutations. Furthermore, cross-species transmission of influenza is frequently preceded by an exchange of gene segments between two viruses, “genetic reassortment,” resulting in even greater genetic variability [14–16]. 

Historically, dogs were not considered to be natural hosts for influenza despite the occasional transmission of viruses to dogs from humans [17, 18], birds [19], and horses [20, 21]. Although, incidents of equine influenza virus (EIV) H3N8 transmission to dogs have been reported in Europe [21], there were no known cases of EIV transmission to dogs in the US until 2004 when a mutated strain of EIV was isolated from racing greyhounds [22, 23] and has been maintained in US dog populations ever since. Amino acid sequence analyses demonstrate that the CIV isolates consistently differ from contemporary equine-lineage H3 viruses (e.g., A/Equine/Kentucky/1/1981, A/Equine/Wisconsin/1/2003, A/Equine/Colorado/10/2007) at five amino acid residues in the hemagglutinin protein (HA), including a tryptophan (W) to leucine (L) substitution at residue 222 located near the receptor binding pocket [22, 23] and seven amino acid mutations within the internal genes [22–24].

Interestingly, results from two recent studies demonstrate that CIV isolates are unable to infect, replicate, and spread among susceptible horses [25, 26]. Moreover, inoculation of horses with canine influenza did not result in clinical disease in either study, indicating the existence of genetic differences in the horse that resulted in an “all or nothing” infection when inoculated with EIV or CIV, respectively. To determine whether contemporary equine viruses are similarly restricted in dogs, we evaluated the infectivity and transmission of a recent EIV isolate in dogs. Additionally, we sought to determine the receptor binding affinity of recent CIV isolates to examine whether the HA W222L mutation has resulted in an alteration in receptor binding affinity of canine isolates.

## 2. Materials/Methods

### 2.1. Influenza Viruses

For the binding assays, A/Equine/Colorado/10/07 (Eq/CO) (H3N8), A/Canine/Colorado/224986/06 (Ca/CO-1) (H3N8), A/Canine/Wyoming/86033/07 (Ca/WY) (H3N8), A/Canine/Colorado/2025974/07 (Ca/CO-2) (H3N8), A/Equine/Kentucky/1/81(H3N8) (Eq/KY; provided as allantoic fluid stock from the University of Wisconsin-Madison's Influenza Virus Repository) (H3N8), and A/Sydney/05/97 (A/Syd; provided as allantoic fluid stocks from the CDC) (H3N2) were cultivated in embryonated hens' eggs or MDCK cells as previously described [27, 28]. Eq/CO and Ca/WY were isolated from horses and dogs, respectively, during recent clinical outbreaks of influenza virus in Colorado and Wyoming regions and were used in the *in vivo* studies as representative EIV and CIV contemporary circulating isolates.

### 2.2. Sequence Analyses

All gene segments from the cultivated Eq/CO and Ca/WY isolates used for inoculation were sequenced and compared using Clustal W (http://www.genome.jp/tools/clustalw/) with their respective parental virus sequences. To confirm the presence of the amino acid differences between the equine and canine H3 viruses, the full-length protein coding regions of the HA genes of Eq/CO, Ca/CO-1, Ca/WY, and Ca/CO-2 were amplified by reverse transcription (RT)-PCR, as previously described [29]. Sequence comparisons of these viruses, as well as published equine and canine influenza virus H3 sequences (obtained from the BLAST database (http://blast.ncbi.nlm.nih.gov)), were made using Clustal W alignments of amino acid residues. Additionally, phylogenetic comparison of CIV and EIV amino acid H3 sequences based on maximum parsimony with 1000 bootstrap replicates was performed using MEGA 5.2.2. 

### 2.3. Equine and Canine Influenza Challenge

Twenty 12-month-old beagle dogs were obtained from a commercial laboratory animal vendor and assigned to one of five groups: two inoculation groups (five dogs/group), one mock-inoculation control group (four dogs/group), or two sentinel groups (three dogs/group). Study groups were housed separately in nearby research facilities. Serum samples from each dog were confirmed to be CIV- and EIV-negative using hemagglutination inhibition (HI) assay before inoculation. The animals were examined prior to inoculation and found to be clinically healthy and in good body condition. Dogs were maintained in accordance with guidelines of Colorado State University Research and Animal Resources Committee. One dog from the Ca/WY inoculation group was withdrawn due to behavioral problems with other dogs in the group. Dogs from inoculated groups were sedated with dexmedetomidine and then infected intranasally and intratracheally with direct deposition of either Ca/WY or Eq/CO 10^7^ 50% tissue culture infectious dose (TCID_50_) onto the nasal and tracheal respiratory epithelium. Sentinel groups were introduced and housed with each infection group two days after inoculation. Before initiation, this study was reviewed and approved for conduct by Colorado State University Institutional Animal Care and Use Committee.

### 2.4. Clinical Scoring

For 14 days following challenge and 21 days after introduction, each inoculated and sentinel dog, respectively, was observed for 20 minutes for clinical signs of infection. For clinical scoring, numbers were assigned based on observations of lethargy, anorexia, sneezing/coughing, respiratory rate, and ocular and nasal discharge, as previously described [26]. Briefly, parameters assessed included general attitude (0 for normal, 1 for lethargic), appetite (0 for normal, 1 for anorexic), cough/sneeze (0 for no cough/sneeze, 1 for less than 3 coughs/sneezes, and 2 for more than 3 coughs/sneezes), and respiratory rate (0 indicating normal respiration, 1 indicating tachypneic, and 2 indicating dyspneic). Discharge was scored as serous (0), mild-moderately mucopurulent (1), or severely mucopurulent (2). The minimum score indicating a healthy animal was 0, and the maximum score indicating a severely ill animal was 8. Rectal temperatures were recorded daily for mean comparison between challenge groups. 

### 2.5. Assessment of Viral Shedding

Nasal swabs were collected daily one day before and for 21 days after inoculation. The swabs were placed in 1 mL of viral transport medium and stored at −80°C until they could be processed for influenza virus isolation. RNA was extracted from 140 *μ*L of viral transport medium using the QIAamp Viral RNA Mini Kit (QIAGEN, Hilden, Germany). Real-time RT-PCR assays were performed using previously established cycling conditions [30]. Briefly, for virus quantification, purified full-length influenza A matrix (M) gene RNA was used as a standard for calibration of the M gene copy number. Each nasal swab sample was run in duplicate. Negative controls included neat transport medium processed as for the nasal swab specimens. The positive controls consisted of 10^1^ to 10^6^ TCID_50_ of Ca/CO-1 in distilled water. 

### 2.6. Serological Analysis

Sera from blood samples taken from inoculated dogs on days 7, 12, and 19 after inoculation and from sentinel dogs on days 7, 12, and 19 after introduction were treated with receptor destroying enzyme prepared from *Vibrio cholera* before they were tested for hemagglutination inhibiting antibodies via HI assay [27]. Briefly, twofold serial dilutions of sera were mixed with four hemagglutination units of Eq/CO and Ca/WY. The assays were developed by adding 0.5% (vol/vol) chicken red blood cells, and the HI antibody titers were read as the reciprocal of the highest dilution causing complete inhibition of hemagglutination.

### 2.7. Virus Binding Affinities

To account for any cell-culture induced mutations, sialic acid (SA) binding affinities of both MDCK cell- and embryonated hens' egg-grown stocks of Ca/CO-1, Ca/WY, Ca/CO-2, and Eq/CO were determined using a solid-phase binding assay [31, 32], with slight modification of the original protocol to equilibrate the viruses assayed to ~20,000 matrix gene copies [33, 34]. The biotinylated glycopolymers tested included Neu5Ac*α*2-3Gal*β*1-4Glc-PAA[1000]-biot (2,3SL), Neu5Ac*α*2-3Gal*β*1-4GlcNAc*β*-PAA[1000]-biot (2,3SLN), Neu5Ac*α*2-6Gal*β*1-4GlcN-PAA[1000]-biot (2,6SL), and Neu5Ac*α*2-6Gal*β*1-4GlcNAc*β*-PAA[1000]-biot (2,6SLN) (Syntesome, Moscow, Russia). All polymers had a molecular weight of 1 megadalton and were diluted 1 : 500 prior to the experiments. Both fetuin-coated and non-fetuin-coated plates were utilized, as conditions for performing the solid-phase binding assay have not yet been described for use with CIV and EIV isolates. Each assay was performed four times in duplicate, including positive (Eq/KY and Eq/CO for SA*α*2,3 binding and A/Syd for SA*α*2,6 binding) and negative (working buffer) controls.

### 2.8. Sialic Acid Staining

To investigate whether the receptor binding specificity of the canine viruses reflect complementary SA receptor expression in the respiratory tract of dogs, we stained sections of donated canine nasal mucosa, larynx, trachea, bronchus, and lung tissue obtained from healthy dogs euthanized for nonrespiratory related problems with SA*α*2,3-gal-specific and SA*α*2,6-gal specific lectins [35, 36]. Fluorescein isothiocyanate (FITC)-labeled *Sambucus nigra* lectin (Vector Laboratories, Burlingame, CA, USA) was used to indicate the presence of SA*α*2,6-gal, while biotinylated *Maackia amurensis* lectin (Vector Laboratories) detected with Alexa Fluor 594-streptavidin complex (Molecular Probes/Invitrogen, Carlsbad, CA, USA) was used to stain SA*α*2,3-gal receptors. Tissues were counterstained with 4,6,-diamidino-2-phenylindole (DAPI). Equine airway tissues collected from respiratory healthy horses euthanized for nonrespiratory related clinical problems were included in the staining procedure to serve as controls, as the pattern of SA expression in the horse trachea has been previously defined [37]. 

### 2.9. Statistical Analyses

To analyze the overall mean differences in the levels of virus nasal shedding, HI antibody titers, clinical scores, and body temperatures between the infected groups and the mock-inoculated controls, we performed generalized estimating equations, as previously described [26]. Briefly, adjusted mean differences were clustered on repeated measures for each outcome. Other outcome variables (except clinical scores, which were ranked prior to analysis) were log transformed to meet the major assumptions of linearity and normality. For data transformation, HI antibody titers and M gene copy numbers with values of zero were converted to one. For the receptor-binding assays, dissociation constants (*K*_*D*_) for viruses were determined by linear regression analysis of Scatchard plots performed using Prism software (GraphPad, La Jolla, CA, USA).

## 3. Results

**Figure 1:**
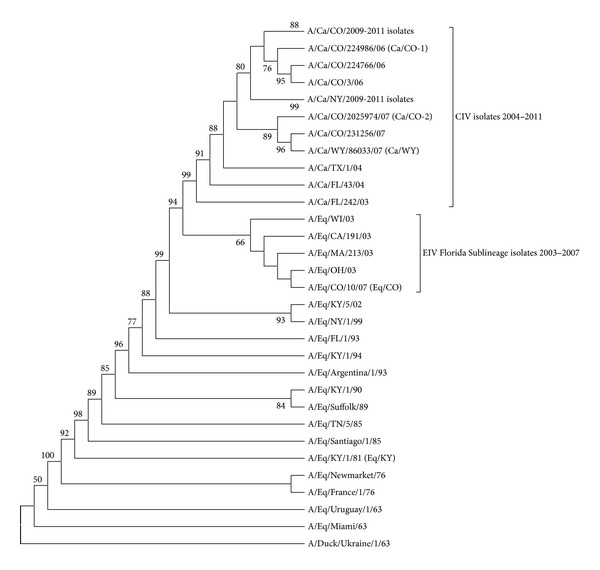
Phylogenetic comparison of canine and equine influenza virus H3 genes. Amino acid analysis was based on maximum parsimony with bootstrap analysis (values with >50% consensus are shown). Challenge and receptor binding assay viruses are in parentheses.

Review of the HA amino acid sequences indicated the five amino acids previously described as possible dog adaptation mutations (N54 K, N83S, W222L, I328T, and N483T) [22, 23] have been conserved in the canine viruses cultivated for and used in this study (Table 1). In contrast, Eq/CO shared the previously described equine H3 consensus sequence [22]. Phylogenetic analyses of the HA genes (Figure 1) demonstrated that Ca/CO-1, Ca/WY, and Ca/CO-2 clustered with the canine isolates and Eq/CO clustered with the contemporary equine viruses, placing them into the previously described distinct canine and equine sublineages of the equine H3 “Florida lineage.” Clustal W gene sequence alignments of the Ca/WY and Eq/CO isolates used for inoculation showed that the amino acid residues of the HA and NA genes did not differ between the challenge and parental viruses (data not shown).

**Figure 2:**
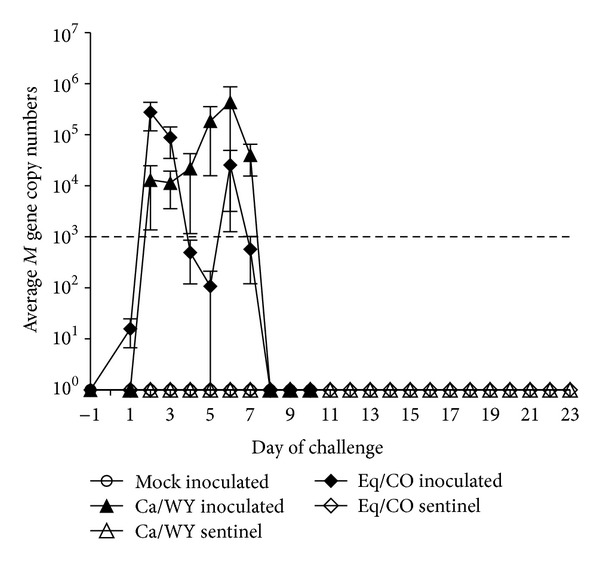
The M gene copy number mean ± SEM of virus shed in nasal passages for mock-inoculated (◯), Ca/WY-inoculated (▲), Ca/WY sentinel (△), Eq/CO-inoculated (◆), and Eq/CO sentinel (*⋄*) dogs. The minimum detection level of the real-time RT-PCR was 1000 M gene copies per reaction corresponding to 10^3^ TCID_50_ of Ca/CO-1 and is represented by the dashed line.

For the *in vivo* challenge, both Ca/WY and Eq/CO inoculated dogs showed little to no signs of clinical disease, despite the evidence that individual dogs were shedding influenza virus (Table 2). Indeed, Eq/Co infected dogs shed virus nasally days 1–7 after inoculation, while Ca/WY infected dogs shed virus days 2–7 after inoculation (Figure 2). All of the mock-inoculated dogs had a clinical score of 0, while the highest clinical score from the Ca/WY group was 3 from a sentinel dog 6 days after being introduced to the inoculated dogs and the highest clinical score from the Eq/CO group was 2 from an inoculated dog on days 6 and 13 after challenge. The Ca/WY sentinel dog with the clinical score of 3 on day 6 after introduction and one Ca/WY-inoculated dog were observed coughing/sneezing and had serous ocular discharge days 4–13 after introduction and days 8–10 after inoculation, respectively. From the Eq/CO group, the dog with the highest clinical score of 2 had periodic anorexia and sneezing between days 3 and 13 after inoculation, and one sentinel showed signs of lethargy on day 3 after introduction and had a clinical score of 1. None of the dogs had a temperature over 103°F during the course of the study.

Although little clinical disease was evident, serologically, 4/4 (100%) of the Ca/WY-inoculated dogs seroconverted by day 12 after inoculation (mean titer 1 : 896 ± 368 SEM), and 2/3 (67%) of the sentinels exposed to the CIV-infected dogs had positive HI assays (mean titer 1 : 1365 ± 557 SEM) by day 12 after introduction (Table 2). Of the Eq/CO inoculated dogs, 80% (4/5) seroconverted (mean titer 1 : 461 ± 152 SEM) by day 12 after inoculation (Table 2), and none of the sentinels had a positive HI assay 7, 12, or 19 days after introduction to the inoculated dogs. Real-time RT-PCR data resemble the HI data for the inoculated dogs from both groups, except for one Eq/CO-inoculated dog who shed virus nasally but never seroconverted and two sentinel dogs from the Ca/WY group who seroconverted without evidence of shedding virus (Table 2). Interestingly, influenza virus was not detected by real time RT-PCR in either the Ca/WY or the Eq/CO sentinel groups. The negative controls did not shed detectable virus, as expected. Generalized estimating equations revealed that, compared to the negative controls, only nasal shedding and antibody titers were significantly higher for both the Ca/WY and Eq/CO-inoculated dogs (Table 3) compared to the mock-inoculated negative controls.

**Figure 3:**
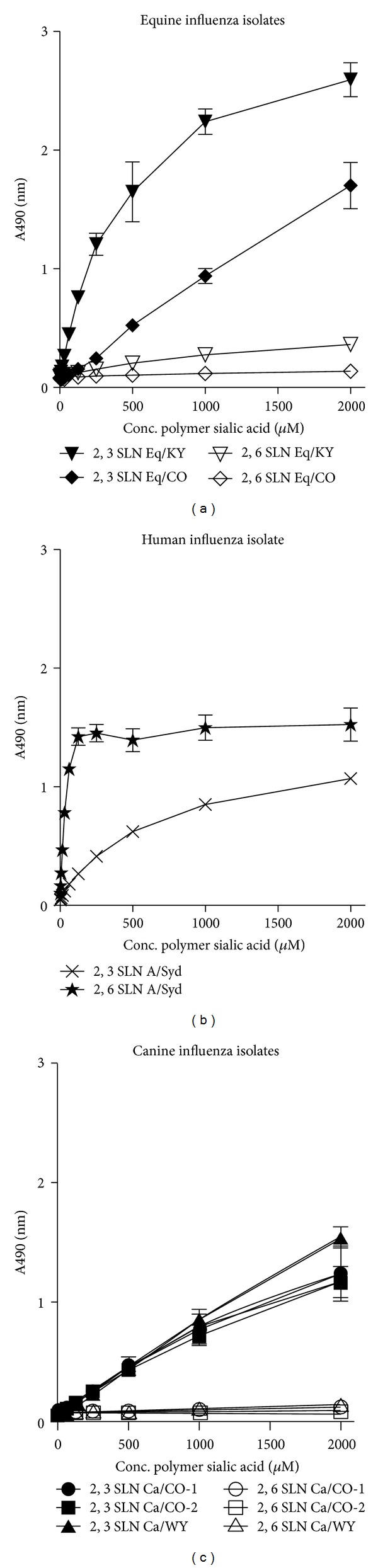
*α*2,3SLN and *α*2,6SLN polymers binding to equine influenza (a), human influenza (b), and canine influenza (c) isolates were determined using a solid-phase binding assay and linear regression analysis.

For the receptor binding affinity experiments, with the exception of A/Syd, which bound only to fetuin-coated plates (as previously described for human influenza viruses [31, 32]), all equine and canine viruses bound only to non-fetuin-coated plates. As the 2,3SL polymer demonstrated similar binding as the 2,3SLN polymer and the 2,6SL polymer is not believed to be a suitable analog for the human influenza virus receptor [38], only the 2,3SLN and 2,6SLN data are presented here for appropriate comparisons. Based on calculated approximate *K*_*D*_ values (where lower values represent higher binding affinity), Eq/CO and Eq/KY demonstrated the anticipated binding preference for SA*α*2,3-gal (2,3SLN) compared to 2,6SLN (Figure 3(a)). In fact, nearly no binding to SA*α*2,6-gal (2,6SLN) was detected. Likewise, the SA*α*2,6-gal control (A/Syd) preferred 2,6SLN as expected (Figure 3(b)). Interestingly, Ca/CO-1, Ca/WY, and Ca/CO-2 showed the same binding preference as the equine viruses, which was characterized by higher affinity for SA*α*2,3-gal (2,3SLN) and only minimal binding to 2,6SLN (Figure 3(c)).  Table 4 lists the *K*_*D*_  values for all the viruses tested with the 2,3SLN and 2,6SLN polymers. Because there was no detectable binding (NDB) to the 2,6SLN polymer for the canine and equine isolates upon linear regression of Scatchard plots, we were unable to determine their *K*_*D*_  values. However, viruses that bound to the 2,3SLN polymer demonstrated low *K*_*D*_  values, indicating that they had high binding affinities for SA*α*2,3-gal. Again, as expected, A/Syd had a human type receptor binding preference with a higher relative affinity for SA*α*2,6-gal than for SA*α*2,3-gal receptor analogues.

**Figure 4:**
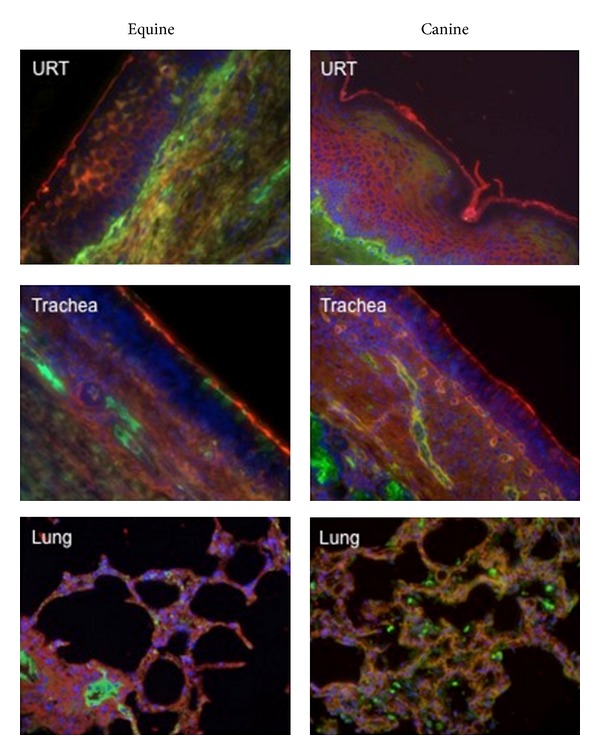
Equine and canine respiratory tissues for the upper respiratory tract (URT) and lower respiratory tract (trachea, lung) stained with lectins specific for sialic acids with *α*2,6- and *α*2,3-linkages. Green staining: reaction with fluorescein isothiocyanate (FITC)-labeled *Sambucus nigra* lectin (Vector Laboratories, Burlingame, CA, USA) indicates the presence of sialic acids linked to galactose by an alpha2,6-linkage (SA*α*2,6-gal). Red staining: reaction with biotinylated *Maackia amurensis* lectin (Vector Laboratories) (detected with Alexa Fluor 594-streptavidin complex; Molecular Probes/Invitrogen, Carlsbad, CA, USA), indicates the presence of SA*α*2,3-gal. Tissues were counterstained with 4,6,-diamidino-2-phenylindole (DAPI).

The staining results revealed that SA*α*2,3-linked receptor (indicated by the red staining in Figure 4) was the predominant receptor expressed on the airway epithelial cells of the upper respiratory tract in both horses and dogs. Furthermore, SA*α*2,3-gal was the primary receptor expressed on the respiratory epithelium throughout the trachea (upper, middle, and lower) and bronchus in both species. This is consistent with the recently published data [21] that also found a predominance of SA*α*2,3-gal in the canine trachea. In contrast, alveoli demonstrated both red and green stainings, suggesting that both SA*α*2,3-gal and SA*α*2,6-gal receptors are expressed deep within the respiratory tract of dogs and horses.

## 4. Discussion

Our results demonstrate that a recent EIV isolate is able to infect and replicate in the canine host. This was evidenced by both CIV- and EIV-inoculated dogs testing positive for antibodies on HI assay and shedding detectable virus on real time RT-PCR. In contrast, recent studies conducted by members of our laboratory [26], as well as others [25], have revealed that CIV has virtually lost the ability to infect, replicate, and spread among susceptible horses. Interestingly, a similar pattern of infectivity and replication has been observed in primary equine and canine respiratory epithelial cells (RECs) inoculated with both CIV and EIV isolates [26]. In these experiments, CIV and EIV isolates were equally able to infect and subsequently replicate in canine RECs, while the EIV isolate was better able to infect and replicate in equine RECs compared to the CIV isolate. Specifically, Quintana et al. (2011) experiments showed that immunocytochemistry staining of Ca/WY nucleoprotein demonstrates a low infectivity phenotype in equine RECs that is paralleled by significantly lower M gene copy numbers in these cells compared to Ca/WY in canine RECs [26]. Taken together, the results from recent studies suggest that there is apparent host range restriction for CIV in horses, which has not been observed for EIV in dogs. Furthermore, equine and canine RECs represent a potential *in vitro* model for determining host range restrictions among EIV and CIV in horses and dogs. Such studies might elucidate how mutated EIV isolates were first able to infect and become transmissible and maintained among dogs. 

Despite infectivity and replication of both isolates in dogs *in vivo*, however, there were some striking differences in kinetics among EIV and CIV infections. As highlighted by Table 2, dogs infected with Eq/CO tended to shed lower influenza virus M gene copy numbers and to shed the highest titers of virus earlier (days 2-3 post inoculation) than dogs that were infected with Ca/CO, who tended to shed the highest numbers of virus relatively later (days 4 to 7 after inoculation). Moreover, one dog that shed the EIV isolate never seroconverted, while none of the EQ/CO sentinels showed evidence of exposure to Eq/CO serologically. In contrast, several dogs exposed to Ca/WY seroconverted without ever showing evidence of infection by viral shedding. These results suggest that, although infection and replication of a contemporary EIV in dogs are experimentally possible, there remains a barrier to transmission among dogs, which is possibly due to differences in EIV and CIV gene segment moieties and/or to the early host immune responses these differences elicit. Again, *in vitro* studies on RECs focused on cytokine and chemokine responses (e.g., TNF-*α*, type 1 interferons, and interleukins) in early EIV and CIV infections might help determine species barriers among the two viruses and help to understand the lack of clinical disease evident in either inoculated group. 

One well-described determinant for influenza virus species-specificity that might explain CIV host range restriction, and one which we wished to examine in these studies, is receptor-binding preference. While evidence suggests that the amino acid sequence of the receptor-binding pocket (formed in part by residues 224 through 228 in H3 viruses) modulates the affinity of influenza viruses for specific SA receptors [39], amino acid residues at other sites in the HA protein may also determine the receptor specificity of influenza viruses. For example, recent research indicates that the amino acid residue 222 in human and swine H1, as well as human and avian H3 viruses, might serve as a key determinant for binding of human receptor analogs by the HA protein [40]. Indeed, early CIV studies postulated that, in dogs, the W222L CIV substitution might play a role in maintenance of influenza by modulating receptor-binding function [22, 23]. 

Interestingly, our solid-phase binding assay results demonstrate that CIV isolates, like EIV isolates, have a higher affinity for SA*α*2,3-gal compared to SA*α*2,6-gal. This preference is mirrored by a predominance of SA*α*2,3-linked receptors in the upper respiratory tract and trachea of dogs. The finding that CIV has a similar overall receptor-binding preference as its equine H3 ancestor might explain the natural transmission of equine influenza viruses to dogs that occurred on at least three separate occasions [20–22]. Our *in vivo* results confirm EIV is still capable of infecting and replicating in dogs. Transmission of that EIV from infected to sentinel dogs would further indicate the ability of EIV to be maintained in dogs. However, our results show no influenza infection in sentinels introduced to the EIV-inoculated group, although a similar study found that dogs could be subclinically infected with EIV when introduced to EIV-infected horses [41]. In spite of these conflicting reports, it still remains unclear why only one of the previous transmission events and none of the experimental studies resulted in the formation of a stable influenza virus lineage within dog populations. 

This lack of dog-to-dog EIV transmission might be explained by the biochemical structure of the SA receptor itself. For example, respiratory epithelial cells in the equine trachea have been found to express mostly *α*2,3-linked *N*-glycolylneuraminic (Neu5Gc) SA receptors rather than *N*-acetylneuraminic (Neu5Ac) SA receptors [17]. Another recent study done by Yamanaka et al. [25], in which solid-phase binding assays were conducted using Neu5Gc and Neu5Ac analogues, suggests that there is an EIV binding preference for the Neu5Gc SA receptor compared to the Neu5Ac SA receptor moiety. However, in the same study, the CIV isolate tested did not appear to have a preference for either the Neu5Ac or Neu5Gc analogue [25], leaving the question of CIV host range restriction in horses unanswered and, therefore, leading us to propose other determinants of CIV species specificity.

Of these potential determinants of host range, the HA protein must still be considered as it also mediates fusion of the viral envelope with the endosomal membrane of the host cell [42]. To mediate fusion, the stalk portion of the HA has to undergo complex refolding [42]. While the specific role of the N83S substitution has yet to be determined, residue 83 is a part of an extension from the central triple-stranded coiled coil (made up of H3 HA residues 76–105), which repositions and exposes the fusion protein when the HA protein is subjected to low pH [43]. The substitution of a positively charged amino acid (asparagine) with a hydrophilic residue (serine) might change the protein structure, possibly altering HA refolding. Similarly, the substitution of threonine (a polar residue) for isoleucine (a nonpolar residue) at the HA cleavage site (residue 328) [43] may have affected host protease-viral protein interactions, thereby modulating efficacy of membrane fusion and viral entry into host cells.

Beyond the HA gene, it is possible that mutations in other RNA segments could account for virus adaptation to dogs. In this regard, it is interesting that sequence alignments revealed three mutations in the PA (residues 33, 388, and 675) and one mutation in the PB2 (residue 374) proteins that consistently differentiate the canine from the equine consensus sequences. As these mutations are located at previously defined functional sites of the PA (PB1 binding site, protease, and cap-dependent ribonuclease regions) and the PB2 (PB1 binding site) [44], it is possible that these substitutions are important for efficient replication of CIV particularly in the canine host. Further reverse genetics and site-directed mutagenesis studies are warranted to address the roles each gene segment plays in CIV species specificity.

## 5. Conclusion

The results of the studies described here contribute to our overall knowledge of EIV and CIV species specificity. We have shown that EIV is still capable of infecting dogs, although we could not show that EIV is transmitted among dogs in an experimental setting. Conversely, as previously reported, the opposite is true for CIV in horses [25, 26]. Additionally, our *in vivo* studies detail similar findings from a previous *in vitro* experiment where both CIV and EIV were able to infect and replicate in primary canine RECs [26]. Combined with data that show a preference of equine RECs for EIV compared to CIV, we believe that we have developed an *in vitro* model using primary canine and equine RECs on which future host range restriction studies can be based. This model might be useful in elucidating host-pathogen interactions that led to the maintenance of influenza viruses in the US dog population.

## Figures and Tables

**Table 1:** Amino acid differences among the hemagglutinin protein of equine and canine influenza viruses. The five amino acids previously identified as EIV H3N8 mutations are in bold.

Virus	HA amino acid position										
	29	54	83	92	118	119	222	261	328	479	483
A/Eq/KY/1/1981	I	**N**	**N**	S	L	E	**W**	R	**I**	G	**N**
A/Eq/WI/1/03	I	**N**	**N**	S	L	E	**W**	K	**I**	G	**N**
A/Eq/CO/10/07	I	**N**	**N**	S	L	E	**W**	K	**I**	G	**N**
A/Ca/FL/242/03	I	**K**	**S**	S	L	E	**L**	K	**T**	G	**T**
A/Ca/FL/43/04	M	**K**	**S**	N	L	E	**L**	K	**T**	G	**T**
A/Ca/CO/224986/06	M	**K**	**S**	N	V	E	**L**	N	**T**	E	**T**
A/Ca/CO/2025974/07	M	**K**	**S**	N	V	K	**L**	N	**T**	E	**T**
A/Ca/WY/86033/07	M	**K**	**S**	N	V	K	**L**	N	**T**	E	**T**

**Table 2:** Nasal shedding of the influenza virus M gene detected on real time RT-PCR and hemagglutination inhibition assay titers to Eq/CO and Ca/WY isolates 12 days after virus inoculation or introduction to inoculated dogs.

Dog ID	Group	Inoculated or sentinel	Highest influenza virus M gene copies (day)	Eq/CO titer	Ca/WY titer
XQV	Ca/WY	Inoculated	296 (day 6)	1 : 512	1 : 256
QZV	Ca/WY	Inoculated	5.74 × 10^4^ (day 7)	1 : 8192	1 : 2048
WOV	Ca/WY	Inoculated	1.74 × 10^6^ (day 6)	1 : 1024	1 : 256
OUV	Ca/WY	Inoculated	8.44 × 10^4^ (day 4)	1 : 2048	1 : 1024
WBV	Ca/WY	Sentinel	0	0	0
SZV	Ca/WY	Sentinel	0	1 : 4096	1 : 2048
KKV	Ca/WY	Sentinel	0	1 : 4096	1 : 2048
LXU	Eq/CO	Inoculated	2.77 × 10^5^ (day 3)	1 : 512	1 : 256
DFS	Eq/CO	Inoculated	6.56 × 10^5^ (day 2)	1 : 1024	1 : 256
VVS	Eq/CO	Inoculated	6.59 × 10^5^ (day 2)	1 : 512	1 : 256
EZS	Eq/CO	Inoculated	1.44 × 10^5^ (day 3)	0	0
DDS	Eq/CO	Inoculated	2.16 × 10^4^ (day 2)	1 : 256	1 : 64
SHU	Eq/CO	Sentinel	0	0	0
EGS	Eq/CO	Sentinel	0	0	0
ALS	Eq/CO	Sentinel	0	0	0

**Table 3:** Statistical analyses of Ca/Wy and Eq/CO challenge groups compared to mock-inoculated negative controls.

	Temperature	Clinical score	Nasal shedding	HIA antibody titer
Ca/WY inoculated	*P* = 0.077	*P* = 0.460	*P* = 0.001*	*P* < 0.001*
Ca/WY sentinel	*P* = 0.608	*P* = 0.130	*P* = 0.704	*P* = 0.185
Eq/CO inoculated	*P* = 0.155	*P* = 0.103	*P* = 0.018*	*P* < 0.001*
Eq/CO sentinel	*P* = 0.691	*P* = 0.474	*P* = 0.704	*P* = 0.175

*Indicates statistical significance at *P* < 0.05.

**Table 4:** Approximate binding affinity of equine and canine influenza viruses.

Virus	2,3SLN polymer		2,6SLN polymer	
	App *K*_*D*_^a^	*R* ^2^ ^b^	App *K*_*D*_	*R* ^2^ ^b^
Eq/KY	11	.96	NDB^c^	—
Eq/CO	5	.99	NDB^c^	—
Ca/CO-1	5	.99	NDB^c^	—
Ca/CO-2	2	.94	NDB^c^	—
Ca/WY	2	.95	NDB^c^	—
A/Syd	139	.97	10	.99

^a^Approximate dissociation constant (*K*_*D*_) values are from one representative experiment. Lower *K*_*D*_ values represent a higher binding affinity for that polymer (*K*_*D*_ is expressed in nM^−1^ sialic acid). Repeated experiments yielded similar results.

^b^ *R* ^2^: coefficient of determination.

^c^NDB: no detectable binding.

## References

[1] Tumpey TM, Basler CF, Aguilar PV (2005). Characterization of the reconstructed 1918 Spanish influenza pandemic virus. *Science*.

[2] Castrucci MR, Kawaoka Y (1993). Biologic importance of neuraminidase stalk length in influenza A virus. *Journal of Virology*.

[3] Hatta M, Halfmann P, Wells K, Kawaoka Y (2002). Human influenza a viral genes responsible for the restriction of its replication in duck intestine. *Virology*.

[4] Dalton RM, Mullin AE, Amorim MJ, Medcalf E, Tiley LS, Digard P (2006). Temperature sensitive influenza a virus genome replication results from low thermal stability of polymerase-cRNA complexes. *Virology Journal*.

[5] Hiromoto Y, Yamazaki Y, Fukushima T (2000). Evolutionary characterization of the six internal genes of H5N1 human influenza A virus. *Journal of General Virology*.

[6] Murphy BR, Buckler-White AJ, London WT, Snyder MH (1989). Characterization of the M protein and nucleoprotein genes of an avian influenza A virus which are involved in host range restriction in monkeys. *Vaccine*.

[7] Scholtissek C, Burger H, Kistner O, Shortridge KF (1985). The nucleoprotein as a possible major factor in determining host specificity of influenza H3N2 viruses. *Virology*.

[8] Snyder MH, London WT, Maassab HF, Chanock RM, Murphy BR (1990). A 36 nucleotide deletion mutation in the coding region of the NS1 gene of an influenza A virus RNA segment 8 specifies a temperature-dependent host range phenotype. *Virus Research*.

[9] Subbarao EK, London W, Murphy BR (1993). A single amino acid in the PB2 gene of influenza A virus is a determinant of host range. *Journal of Virology*.

[10] Tian SF, Buckler-White AJ, London WT (1985). Nucleoprotein and membrane protein genes are associated with restriction of replication of influenza A/mallard/NY/78 virus and its reassortants in squirrel monkey respiratory tract. *Journal of Virology*.

[11] Bender C, Hall H, Huang J (1999). Characterization of the surface proteins of influenza A (H5N1) viruses isolated from humans in 1997-1998. *Virology*.

[12] Suarez DL, Perdue ML, Cox N (1998). Comparisons of highly virulent H5N1 influenza A viruses isolated from humans and chickens from Hong Kong. *Journal of Virology*.

[13] Zhou NN, Shortridge KF, Claas ECJ, Krauss SL, Webster RG (1999). Rapid evolution of H5N1 influenza viruses in chickens in Hong Kong. *Journal of Virology*.

[14] Fang R, Jou WM, Huylebroeck D (1981). Complete structure of A/duck/Ukraine/63 influenza hemagglutinin gene: animal virus as progenitor of human H3 Hong Kong 1968 influenza hemagglutinin. *Cell*.

[15] Gething MJ, Bye J, Skehel J, Waterfield M (1980). Cloning and DNA sequence of double-stranded copies of haemagglutinin genes from H2 and H3 strains elucidates antigenic shift and drift in human influenza virus. *Nature*.

[16] Kawaoka Y, Krauss S, Webster RG (1989). Avian-to-human transmission of the PB1 gene of influenza A viruses in the 1957 and 1968 pandemics. *Journal of Virology*.

[17] Chang CP, New AE, Taylor JF, Chiang HS (1976). Influenza virus isolations from dogs during a human epidemic in Taiwan. *International Journal of Zoonoses*.

[18] Pysina TV, Surin NG (1972). Isolation from dogs of an influenza virus similar to A2 (Hong Kong)68. *Voprosy Virusologii*.

[19] Song D, Kang B, Lee C (2008). Transmission of avian influenza virus (H3N2) to dogs. *Emerging Infectious Diseases*.

[20] Newton R, Cooke A, Elton D (2007). Canine influenza virus: cross-species transmission from horses. *Veterinary Record*.

[21] Daly JM, Blunden AS, MacRae S (2008). Transmission of equine influenza virus to english foxhounds. *Emerging Infectious Diseases*.

[22] Crawford PC, Dubovi EJ, Castleman WL (2005). Epidemiology: transmission of equine influenza virus to dogs. *Science*.

[23] Payungporn S, Crawford PC, Kouo TS (2008). Influenza A virus (H3N8) in dogs with respiratory disease, Florida. *Emerging Infectious Diseases*.

[24] Dillion S, Spindel ME, Landolt GA Genetic characterization of canine and equine H3N8 influenza viruses isolated in Colorado and Wyoming between 2006 and 2007.

[25] Yamanaka T, Tsujimura K, Kondo T (2010). Infectivity and pathogenicity of canine H3N8 influenza A virus in horses. *Influenza and other Respiratory Viruses*.

[26] Quintana AM, Hussey SB, Burr EC (2011). Evaluation of infectivity of a canine lineage H3N8 influenza a virus in ponies and in primary equine respiratory epithelial cells. *American Journal of Veterinary Research*.

[27] Landolt GA, Karasin AI, Phillips L, Olsen CW (2003). Comparison of the pathogenesis of two genetically different H3N2 influenza a viruses in pigs. *Journal of Clinical Microbiology*.

[28] Lunn DP, Soboll G, Schram BR (1999). Antibody responses to DNA vaccination of horses using the influenza virus hemagglutinin gene. *Vaccine*.

[29] Karasin AI, Brown IH, Carman S, Olsen CW (2000). Isolation and characterization of H4N6 avian influenza viruses from pigs with pneumonia in Canada. *Journal of Virology*.

[30] Landolt GA, Karasin AI, Hofer C, Mahaney J, Svaren J, Olsen CW (2005). Use of real-time reverse transcriptase polymerase chain reaction assay and cell culture methods for detection of swine influenza A viruses. *American Journal of Veterinary Research*.

[31] Gambarayan AS, Matrosovich MN (1992). A solid-phase enzyme-linked assay for influenza virus receptor-binding activity. *Journal of Virological Methods*.

[32] Matrosovich M, Tuzikov A, Bovin N (2000). Early alterations of the receptor-binding properties of H1, H2, and H3 avian influenza virus hemagglutinins after their introduction into mammals. *Journal of Virology*.

[33] Spindel MF, Dillon S, Lunn KF, Landolt GA (2007). Detection and quantification of canine influenza virus by one-step real-time reverse transcription PCR. *Journal of Veterinary Internal Medicine*.

[34] Bateman AC, Busch MG, Karasin AI, Bovin N, Olsen CW (2008). Amino acid 226 in the hemagglutinin of H4N6 influenza virus determines binding affinity for *α*2,6-linked sialic acid and infectivity levels in primary swine and human respiratory epithelial cells. *Journal of Virology*.

[35] Hall JS, Bentler KT, Landolt G (2008). Influenza infection in wild raccoons. *Emerging Infectious Diseases*.

[36] Shinya K, Ebina M, Yamada S, Ono M, Kasai N, Kawaoka Y (2006). Influenza virus receptors in the human airway. *Nature*.

[37] Suzuki Y, Ito T, Suzuki T (2000). Sialic acid species as a determinant of the host range of influenza A viruses. *Journal of Virology*.

[38] Eisen MB, Sabesan S, Skehel JJ, Wiley DC (1997). Binding of the influenza A virus to cell-surface receptors: structures of five hemagglutinin-sialyloligosaccharide complexes determined by X-ray crystallography. *Virology*.

[39] Weis W, Brown JH, Cusack S, Paulson JC, Skehel JJ, Wiley DC (1988). Structure of the influenza virus haemagglutinin complexed with its receptor, sialic acid. *Nature*.

[40] Wang Q, Tian X, Chen X, Ma J (2007). Structural basis for receptor specificity of influenza B virus hemagglutinin. *Proceedings of the National Academy of Sciences of the United States of America*.

[41] Yamanaka T, Nemoto M, Tsujimura K, Kondo T, Matsumura T (2009). Interspecies transmission of equine influenza virus (H3N8) to dogs by close contact with experimentally infected horses. *Veterinary Microbiology*.

[42] Wright PF, Neumann G, Kawaoka Y, Knipe DM, Howley PM (2007). Orthomyxoviruses. *Fields Virology*.

[43] Skehel JJ, Wiley DC (2000). Receptor binding and membrane fusion in virus entry: the influenza hemagglutinin. *Annual Review of Biochemistry*.

[44] Masunaga K, Mizumoto K, Kato H, Ishihama A, Toyoda T (1999). Molecular mapping of influenza virus RNA polymerase by site-specific antibodies. *Virology*.

